# Thyroid-Induced Mania: A Case Highlighting the Role of Organic Etiologies in Psychiatric Presentations

**DOI:** 10.1192/j.eurpsy.2026.11048

**Published:** 2026-07-31

**Authors:** M. Mouldi, O. Ghorbel, H. Ghabi, M. Karoui, H. Nefzi, R. Kamoun, F. Ellouze

**Affiliations:** Department of Psychiatry G, Razi Hospital, Manouba, Tunisia

## Abstract

**Introduction:**

Endocrine pathologies, particularly thyroid dysfunction, are known to precipitate a wide spectrum of neuropsychiatric manifestations. Among these, hyperthyroidism has been implicated in the genesis of acute manic episodes.

**Objectives:**

We present a case underscoring the critical necessity of screening for organic causes, specifically thyroid dysfunction, in patients presenting with symptoms suggestive of a primary bipolar disorder.

**Methods:**

The case involves a 31-year-old male with a family history of thyroid disease but no personal psychiatric or thyroid history. He was hospitalized for severe psychomotor agitation and hyperactivity, necessitating physical restraint. Laboratory investigations revealed suppressed Thyroid-Stimulating Hormone (TSH) and elevated Free Thyroxine (FT4) levels. An endocrinology consult resulted in the initiation of methimazole (30mg/day) and propranolol (80mg/day).

**Results:**

The patient’s presentation demonstrates the potent role of hyperthyroidism in mimicking a primary manic episode. This finding stresses the clinical imperative to identify and address underlying medical conditions that can manifest as psychiatric illness. Prompt diagnosis and treatment of the organic cause are essential for effective symptom resolution and improved patient prognosis.

**Conclusions:**

This report reinforces the need for a high index of suspicion for medical etiologies, such as hyperthyroidism, in patients presenting with acute mania. Systematic screening for these conditions allows clinicians to administer targeted treatment, thereby optimizing patient care and outcomes.

**Disclosure of Interest:**

None Declared

